# Hydrothermal Treatment Effect on Antioxidant Activity and Polyphenols Concentration and Profile of *Brussels sprouts* (*Brassica oleracea* var. *gemmifera*) in an In Vitro Simulated Gastrointestinal Digestion Model

**DOI:** 10.3390/antiox11030446

**Published:** 2022-02-23

**Authors:** Joanna Doniec, Adam Florkiewicz, Kinga Dziadek, Agnieszka Filipiak-Florkiewicz

**Affiliations:** 1 Department of Plant Products Technology and Nutrition Hygiene, Faculty of Food Technology, University of Agriculture in Krakow, 122 Balicka St., 30-149 Krakow, Poland; agnieszka.filipiak-florkiewicz@urk.edu.pl; 2 Department of Food Analysis and Quality Assessment, Faculty of Food Technology, University of Agriculture in Krakow, 122 Balicka St., 30-149 Krakow, Poland; adam.florkiewicz@urk.edu.pl; 3 Department of Human Nutrition and Dietetics, Faculty of Food Technology, University of Agriculture in Krakow, 122 Balicka St., 31-149 Krakow, Poland; kinga.dziadek@urk.edu.pl

**Keywords:** *Brussels sprouts*, boiling, steaming, sous-vide, polyphenols, antioxidant activity, in vitro digestion

## Abstract

*Brussels sprouts* are a source of polyphenolic compounds. However, their concentration is affected by many factors depending on the plant material, hydrothermal treatment methods and digestion in the gastrointestinal tract. The aim of this study was to determine the effect of hydrothermal treatment on the antioxidant activity, concentration and profile of polyphenols of *Brassica oleracea* var. *gemmifera* in an in vitro simulated gastrointestinal digestion model. The study showed a significant effect of the type of hydrothermal treatment on total polyphenol concentration, polyphenolic acid profile, flavonoid content and antioxidant activity. Traditional boiling in water was the least effective type of hydrothermal treatment with respect to bioactive components of *Brussels sprouts*. Sous-vide was the most effective hydrothermal treatment in terms of retention of polyphenolic compounds and high antioxidant activity, thus providing a better alternative to steam cooking. Using an in vitro model, a significant difference was demonstrated between the concentration of bioavailable polyphenolic compounds and the polyphenol content of the plant material before digestion. The influence of the type of hydrothermal treatment used on the concentration of bioavailable polyphenolic compounds was maintained in relation to material not subjected to in vitro digestion (except for antioxidant activity).

## 1. Introduction

*Brassicaceae* vegetables are consumed worldwide and appreciated for their taste, aroma and nutritional value. The high concentration of secondary metabolites and bioactive compounds (including polyphenols, phenolics, phenolic acids, carotenoids, flavonoids, alkaloids, glucosinolates, tocopherols and others) provides *Brassicaceae* species with pro-health effects. Numerous studies indicate a protective effect at high consumption of cruciferous vegetables against type II diabetes, obesity, cardiovascular diseases, osteoporosis and cancer. Additionally, high consumption of *Brassicaceae* species is also correlated with antimicrobial activity and broad antioxidant capacity. Plant polyphenols present in *Brassicaceae* vegetables show anti-inflammatory, antioxidant, cardioprotective, antimicrobial and anticancer activities [1]. When analysing the pro-health effects of polyphenols, an important issue to consider is the level of consumption of these compounds necessary to induce therapeutic functions on the human body, which is influenced by many factors [2]. In addition to cultivation and climatic conditions, soil type, post-harvest treatment methods and storage, the content, composition and bioavailability of polyphenolic compounds in vegetables are also affected by the thermal treatment methods preceding their consumption. The type of hydrothermal treatment chosen affects the concentration of bioactive compounds in the raw plant material not only through direct temperature action, but also through the loss of polyphenols from plant tissues by leaching into the medium in which they are prepared. Researchers have shown that there are also changes in the polyphenol profile of cruciferous vegetables during thermal treatments which apply direct contact of vegetables with water medium (blanching and boiling). It is mainly a reduction of polyphenolic acid content due to leaching that has been detected [3,4]. The sous-vide method, as an alternative to the conventional preparation of plant raw materials by vacuum packaging in plastic vacuumized pouches and hydrothermal treatment with strict control of temperature conditions, can be characterized by higher concentration of bioactive compounds compared with plant raw materials subjected to conventional hydrothermal treatment, thus retaining higher nutritional value of the original product. Analysis of the effect of hydrothermal treatment is crucial in the case of *Brassicaceae* vegetables, including the *Brussels sprouts* *Brassica oleracea* var. *gemmifera* analysed within this work, due to how their consumption is normally preceded by a thermal process rather than simply consumed in their raw state [5].

However, before the nutrients and bioactive compounds can be used in the body, they must first be digested in the gastrointestinal tract in order to be absorbed and assimilated in a suitable form. Studies that are based solely on determining the concentration of bioactive components in the plant material do not therefore provide sufficient information about the bioavailable content of these compounds as they reach the systemic circulation system and become available for use at the target site of the human body. Analyses involving the use of an in vitro digestion model provide more detailed data on the concentration of the studied components after ingestion and digestion within the stomach and intestines in vivo, in simulated in vitro laboratory conditions [6]. They enable a determination of the influence of digestive conditions on food composition, an understanding of the structure of food products and a determination of nutritional value based on the concentrations of nutrients and bioactive components available for absorption and utilisation by the human body [6,7,8]. Research on the antioxidant activity, concentration and profile of polyphenolic compounds of raw and heat-treated *Brussels sprouts* has focused only on the analysis of the plant material. To the authors’ knowledge, there have been no reports on the antioxidant activity, bioavailable concentration and profile of polyphenolic compounds of *Brassica oleracea* var. *gemmifera* after in vitro digestion.

The aim of this work was to determine the effect of hydrothermal treatment on the antioxidant activity and polyphenol concentration and profile of *Brussels sprouts* (*Brassica oleracea* var. *gemmifera*) in an in vitro simulated gastrointestinal digestion model.

## 2. Materials and Methods

### 2.1. Experimental Material

The study object was *Brussels sprouts* (*Brassica oleracea* var. *gemmifera*) purchased in 2019 from a local supermarket (Cracow, Poland). *Brussels sprouts* (2 kg fresh weight of plant material, in three replicates) were cleaned and then divided into 4 sub-samples. One sub-sample was left raw—the plant material not subjected to thermal treatment (n = 3). Three sub-samples were subjected to different hydrothermal treatments: steaming (n = 3), traditional boiling in water (n = 3) and sous-vide (n = 3). Parameters (time, temperature, water ratio) of the hydrothermal processes have been optimized in previous studies [5,8]. Steaming was conducted in a Retigo Orange Version 6 × GN1/1|O 611 in combi steamer (Retigo, Rožnov pod Radhoštěm, Czech Republic) at 100 °C for 7 min. Hydrothermal treatment by means of traditional boiling in water was conducted for a period of 15 min from the boiling of the water until the level of consumable softness was reached at 98 ± 1 °C using stainless steel cookware on an induction hob (Hendi, Hamburg, Germany). The following plant raw material to water ratio of 1:3 (*w*:*v*) was maintained during the process (material was covered as it was boiled). Before hydrothermal sous-vide treatment, the plant material samples were packed in vacuum bags (Hendi, Hamburg, Germany, ca number 970683) using a VBN-4 (RM Gastro, Veselí nad Lužnicí, Czech Republic) and the sous-vide system model 225,448 (Hendi, Hamburg, Germany) was used. The process lasted 50 min at constant temperature control maintained at 90 °C. The methanolic extracts for the determination of total polyphenols, ABTS and DPPH antioxidant activity and polyphenol profile were prepared from fresh plant material and dried plant material after thermal treatments. In vitro digestion was also performed on the fresh plant material and drained plant material after thermal treatments. The extracts and samples after in vitro digestion for the analyses were stored at −22 ± 1 °C in tightly closed, airtight containers.

### 2.2. In Vitro Digestion

In vitro digestion was performed following the methodology in ref. [9], with some modification [10]. Homogenised plant material (0.5 g) was placed in syringes, distilled water was added at a volume of 1 mL and mixed. Subsequently, 0.5 M HCl was added at a volume sufficient to reach pH = 2 and 0.2 mL pepsin solution (activity 3850 U/mg; concentration 6 mg/mL; company Sigma-Aldrich P6887, Saint Louis, MO, USA); dissolved in 0.1 M HCl) was added. This was then supplemented with redistilled water and incubated at 37 °C for 2 h (gastric in vitro digestion fragment). Then, 1 M NaHCO_3_ was added to an amount providing pH = 7 and 0.5 mL of pancreatin and bile solutions were added (pancreatin and bile concentrations of 9 mg/mL and 90 mg/mL, respectively; Sigma bile (B8631) dissolved in 0.1 M NaHCO_3_ and Sigma pancreatin (P7545)). This was again supplemented with redistilled water (intestinal in vitro digestion fragment). The contents of the syringe were mixed and transferred to dialysis bags (dialysis tubing cellulose membrane Sigma-Aldrich D9777-100FT, Saint Louis, MO, USA), which were closed and placed in the imidazole buffer solution (3.4 g of Sigma imidazole (792527) dissolved in 250 mL of redistilled water, adjusted to pH 7.0; with 11.688 g of NaCl anhydrous and supplemented with redistilled water to 2 dm^3^). The flasks were placed in a shaking water bath (GFL 1092, 40 rpm/min) and incubated at 37 °C for 2 h. The resulting dialysate was used for further analyses (in the liquid form without freeze-drying).

### 2.3. Preparation of Methanolic Extracts

Methanol extracts for the determination of total polyphenols, ABTS and DPPH antioxidant activity were prepared from the fresh plant material and plant material after hydrothermal treatment. The plant material (1 g) was weighed into an Erlenmeyer flask and 80 mL of 70% methanol (POCH, Gliwice, Poland) was added. The samples were extracted at room temperature (25–30 °C) using a laboratory shaker at limited light for 2 h (Elpin Plus, type 357, Lubawa, Poland). The extracts were then centrifuged for 15 min at 1500 rpm. (MPW 352R Centrifuge, MPW Med. Instruments, Warsaw, Poland). The obtained supernatant was stored at −22 ± 1 °C in hermetically sealed containers used for further analyses [11]. 

### 2.4. Determination of Total Polyphenols Concentration

The total polyphenol content was determined in methanolic extracts of the raw and hydrothermally-treated plant samples and intestinal dialysates made by in vitro digestion of the raw and hydrothermally-treated plant samples. Folin–Ciocalteu reagent (Sigma-Aldrich, Saint Louis, MO, USA) was used for this determination. The results were expressed as gallic acid equivalent (mg/100 g fresh sample) [12].

### 2.5. Determination of Antioxidant Activity

The antioxidant activity was measured in methanolic extracts of the raw and hydrothermally-treated plant samples and in the intestinal preparations (solutions obtained by in vitro digestion of the raw and hydrothermally-treated plant samples). In order to determine the antioxidant activity, two methods were used—with the DPPH and with ABTS free radicals. The determination of the antioxidant activity of the samples using ABTS^●+^ free radicals (2,2′-Azino-bis (3-ethylbenzthiazoline-6-sulfonic acid) was based on the methodology of ref. [13]. The results obtained were compared with the concentration–response curve and expressed as micromoles of Trolox equivalent per gram weight (TEAC) of the samples. The determination of the antioxidant activity of the samples with the DPPH· radicals (2,2-diphenyl-1-picryl-hydrazyl-hydrate) was performed based on the methodology of ref. [14]. The results obtained were compared with the concentration–response curve and expressed as micromoles of Trolox equivalent per gram weight (TEAC) of the samples.

### 2.6. Determination of Polyphenolic Acid Profile

#### 2.6.1. Preparation of Extracts

To determine the polyphenolic acid profile of the raw and hydrothermally-treated plant samples, methanol-acetone extracts were prepared by a two-stage extraction. The first extraction step was performed with 80% methanol with 0.16 mol/L HCl and the second with 70% acetone. The supernatants from the two-stage extraction were combined and the resulting solution was used to determine the polyphenolic acid profile (extracts were stored at −20 °C until determination) [15]. 

#### 2.6.2. Chromatographic Analysis of Polyphenolic Acid Profile

In order to determine the profile of polyphenolic acid profile in the raw and hydrothermally-treated plant samples, hydrolysis was performed prior to the chromatographic analysis according to the methodology of ref. [16]. Briefly, 10 mL of sample extract, or 10 mL of solutions obtained during in vitro digestion, was mixed with 90 mL of 2 mol/L NaOH (with addition of 1% ascorbic acid and 10 mmol/L ethylenediaminetetraacetic acid). Solutions were then incubated at 30 °C for 30 min. After incubation pH of solution was adjusted to 3 with 4 mol/L HCl, and then saturated with NaCl. A triple extraction was performed using ethyl acetate (volume proportion of the aqueous extract to organic solution was 2:1). Ethyl acetate fractions were then collected and evaporated using a rotary evaporator in 35 °C, under reduced pressure (RE-100-PRO, ChemLand, Stargard, Poland). Obtained dry residue was finally dissolved with methanol into a volume of 5 mL. The solutions were filtered with Millex-LCR filters (Millipore, Burlington, MA, USA) with 0.45 μm membrane pore diameter before chromatographic analysis. The quantitative and qualitative analysis of polyphenolic compounds was performed following the methodology of ref. [17] using high-performance liquid chromatography (Prominence-i LC-2030C 3D Plus system, Shimadzu, Kyoto, Japan) with a diode array detector (DAD). A Luna Omega 5 µm Polar C18, 100 A, 250 × 10 mm column (Phenomenex, Torrance, CA, USA) was used for the determination and was operated at 40 °C. The detection of 4-hydroxybenzoic acid, myricetin, quercetin, luteolin and isorhamnetin was undertaken at 254 nm, rutin at 256 nm, vanillic acid at 260 nm, kaempferol at 264 nm, apigenin and acacetin at 267 nm, gallic acid at 271 nm, hispidulin at 273 nm, syringic acid at 274 nm, catechin and epicatechin at 278 nm, naringin and carnosol at 283 nm, hesperidin and carnosic acid at 284 nm, *p*-coumaric acid at 310 nm, caffeic acid, ferulic acid and sinapinic acid at 323 nm, chlorogenic acid at 326 nm and rosmarinic acid at 329 nm. The gradient program mode was at a flow rate of 1.2 mL/min. The mobile phases consisted of two solvents: A—0.1% formic acid in water (*v*/*v*) and B—0.1% formic acid in methanol (*v*/*v*). Chromatographic analysis was carried out using a gradient program: from 20% to 40% B in 10 min, 40% B for 10 min, from 40% to 50% B in 10 min, from 50% to 60% B in 5 min, 60% B for 5 min, from 60 to 70% B in 5 min, from 70% to 90% B in 5 min, 90% B for 5 min, from 90% to 20% B (the initial condition) in 1 min and 20% B for 4 min (total time—60 min). The amount of individual phenols was determined based on calibration curves, executed in triplicate and plotted individually for each standard. A stock standard solution (100 mg/L) of each polyphenolic compound was prepared in 0.1% formic acid in 70% methanol (*v*/*v*) (POCH, Poland). The calibration curves of the polyphenol standards were made by dilution of stock standard solutions in 0.1% formic acid in 70% methanol (*v*/*v*) to yield 1.56–25 mg/L (5 points). All the solutions were filtered through a 0.22 µm filter.

### 2.7. Statistical Analysis

The analyses and thermal treatments were performed in triplicate. The results were expressed as mean value ± standard deviation. The results were statistically processed by the multivariate analysis of variance (ANOVA). The significance of the differences between the mean values was determined by Tukey’s HSD post-hoc test at a significance level of *p* ≤ 0.05. The Statistica 13 software package (StatSoft Inc., Tulsa, OK, USA) was used for the calculations.

## 3. Results and Discussion

### 3.1. Polyphenols Concentration of Brussels sprouts Raw and Subjected to Hydrothemal Treatments before and after In Vitro Digestion

**Figure 1:**
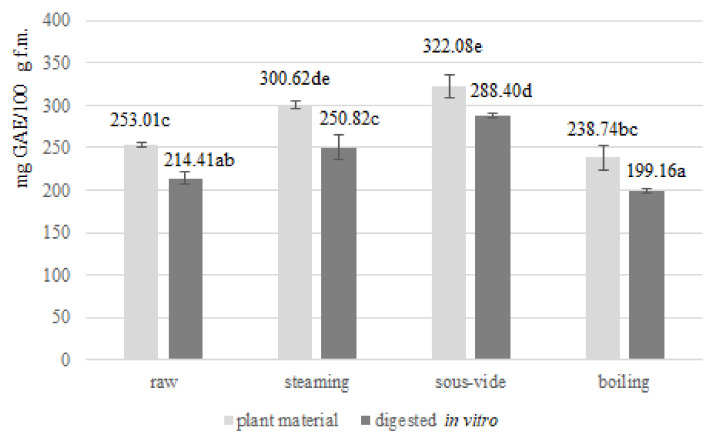
Polyphenols concentration (mg GAE/100 g f.m.) in raw and thermally-treated *Brussels sprouts* before and after in vitro digestion. Means followed by the same letter (a,b,c,d,e) are not significantly different (*p* ≤ 0.05); f.m.—fresh matter.

Polyphenols have a chemical structure that enables the capture and neutralisation of free radicals. This activity is associated with their anti-aging, anti-diabetic, anti-cancer, cardioprotective and neuroprotective effects [18]. Total polyphenols concentration was determined spectrophotometrically by measuring the absorbance of a coloured complex of polyphenolic compounds with the Folin–Ciocalteu reagent. The concentration of total polyphenols in raw and thermally-treated *Brussels sprouts* before and after in vitro digestion is presented in the graph in Figure 1. The raw *Brussels sprouts* had a total polyphenol concentration of 253.01 ± 2.66 (mg GAE/100 g fresh material). A comparable value was determined in *Brassica oleracea* var. *gemmifera* by other authors (230.66 ± 2.85 mg GAE/100 g f.m.), while broccoli, white rose cauliflower and Romanesco type cauliflower had lower total polyphenol content than *Brussels sprouts* (range 74.61–136.54 mg GAE/100 g f.m.) [5]. Furthermore, lower concentration (range 41.8–160 mg GAE/100 g f.m.) of polyphenols than in the examined *Brussels sprouts* was detected by other authors in *Brassica* vegetables: Swede (*Brassica napus* var. *napobrassica,* var. Vige), Kailan-hybrid broccoli (*Brassica oleracea* Italica Group × Albogla- bra Group, cv. Bimi^®^); broccoli (*Brassica oleracea* L. cv. Parthenon); broccoli cv. Marathon (*Brassica oleracea* var. *italica*), broccoli cv. Parthenon (*Brassica oleracea* var. italica), broccoli cv. Graffiti (*Brassica oleracea* var. *botrytis*), broccoli cv. Pastoret (*Brassica oleracea* var. *botrytis*), Espigal del Garraf (*Brassica oleracea* var. *acephala*), and kale cv. Crispa (*Brassica oleracea* var. *acephala*) [18,19,20,21]. These differences can be dictated both by the different species of *Brassica* vegetables that were analysed as well as by the divergences in their cultivation methods and their different countries of origin. 

Steaming and sous-vide increased the concentration of total polyphenols, while traditional cooking in water contributed to a reduction in their content. The increase may be due to damage to cell walls and membranes during thermal treatment, thus increasing the pool of polyphenols available for extraction and thus for determination. In addition, compounds such as phenolic sugar glycosidic bounds, which have a higher affinity for the Foline–Ciocalteu reagent, are released during hydrothermal treatments of vegetables. As a result of the high temperature that accompanies thermal treatment, the polyphenol oxidase enzyme, an enzyme responsible for the degradation of polyphenols, is also inactivated, thus its inhibition also increases the pool of polyphenols. During traditional cooking in water, the application of high temperatures makes the polyphenolic compounds available but they are also more easily leached out to the cooking water. These results are consistent with data from other authors [21,22]. However, in opposition, there are also studies in which the polyphenol content following thermal treatment of *Brassica* vegetables was lower than that of untreated plant material [18,19,20]. These differences may be dictated both by the different species of Brassica vegetables analysed as well as by divergences in their cultivation methods, their different countries of origin and the divergent conditions of the hydrothermal treatments applied. Additionally, they also result from the structure of the individual *Brassica* vegetables, *Brussels sprouts*, having a compact structure, are characterised by a higher retention rate of nutrients and bioactive components than broccoli and cauliflower, which have larger contact surfaces with the aqueous medium due to their more staggered structure. Among the thermal treatments analysed in our study, the sous-vide treatment technique, due to the use of vacuum packaging limiting the loss of nutrients and bioactive compounds through leaching into the aqueous medium, contributed to the highest polyphenol levels in *Brassica oleracea* var. *gemmifera* (*p* ≤ 0.05). A similar correlation of the positive effect of sous-vide in relation to the concentration of total polyphenols was also presented by other authors [5,20]. Also ref. [19] identified boil-in-bag and sous-vide as methods that would allow better retention of polyphenolic compounds due to vacuum packaging than conventional methods for Swede (*Brassica napus* var. *napobrassica*, var. Vige). Other authors [21,22] have presented grilling and the use of microwaves as those methods for which the total polyphenol content was the highest, a relationship powered by the short processing time of these methods compared with others. In contrast, ref. [18] observed the divergent effect of the treatment methods in relation to the morphological parts of the plant, with the stems of broccoli retaining the highest proportion of polyphenols in the case of sous-vide treatment, while the processing of the inflorescences of broccoli with this treatment was associated with greater losses than with the other methods, something which is directly related to the difference in the structure of the different morphological parts of the plants. 

For all treatment conditions and the raw sample, the concentration of polyphenols after in vitro digestion was significantly reduced compared with the plant material before digestion. The highest concentration of polyphenols detected in samples both prior and after in vitro digestion were those involved in the sous-vide treatment, while the lowest were in those involved in traditional boiling in water. The concentration of bioavailable polyphenols after the sous-vide technique was preserved at a higher level than in the case of the raw plant material after digestion. Thus confirming that the sous-vide method, through the use of vacuum packaging and controlled process temperature conditions, is, among the hydrothermal treatment techniques used, the most effective in relation to the preservation of bioavailable polyphenolic compounds of *Brussels sprouts*. Polyphenols in food occur in the form of glycosides, polymers and esters. These compounds cannot be absorbed by the human digestive system in their native biochemical form and require hydrolysis with the participation of digestive system enzymes and intestinal microflora. Approximately 10% of polyphenolic compounds supplied with food do not undergo hydrolysis and remain in an unchanged form in the food matrix, thus not being used by the human body [23]. These data are in accordance with those obtained in our own research. We detected a lower concentration of bioavailable polyphenols after in vitro digestion in comparison to the content of polyphenolic compounds present in the plant material prior to digestion. However, the authors also based their study on an in vitro model that showed a high stability of polyphenolic compounds after the digestion stage, simulating in vivo digestion within the oral cavity and stomach. Studying the effect of in vitro digestion on the bioavailability of rutin, caffeic and rosmarinic acid in three in vitro models (centrifugation, filtration and dialysis), they showed no significant differences between the initial values of the studied compounds collected for digestion and the concentration of bioavailable compounds analysed after the gastric stage of digestion. Thus, showing that the digestion conditions used did not significantly affect the stability of rutin, caffeic and rosmarinic acid [24]. The protective effect of low pH during the in vitro gastric digestion step against polyphenolic compounds was also confirmed in another study [25]. High stability of total polyphenols was also demonstrated by in vitro digestion of grape polyphenols in the case of the gastric digestion section (increase in concentration). Whereas, and similar to our own study, a decrease in the concentration of bioavailable polyphenolic compounds was noted in the case of further intestinal in vitro digestion requiring an alkaline environment. Thus indicating that the pH conditions of digestion have a significant effect on the concentration of bioavailable compounds [26]. Furthermore, fruits, leaves and branches of blackthorn (*Prunus spinosa* L.) have, similar to our own study, demonstrated that polyphenolic compounds are sensitive to slightly alkaline intestinal environment in vitro [27]. Similarly, bamboo leaves soup recorded a 1.64% increase in TPC during the gastric in vitro digestion stage and a 19.97% decrease during the intestinal in vitro digestion stage [28]. Oppositely, there are also works in which the total concentration of polyphenolic compounds was also reduced during the gastric digestion stage for plant-processing by-products of black carrot, aqueous infusions from *Crithmum Maritimum* L. and *Capparis Spinosa* L. and yerba mate, chamomile tea, coffee-like substitutes and coffee blend (65% roasted: 35% green). This decrease in bioactive compounds was further exacerbated in the subsequent intestinal part of the in vitro digestion [29,30,31]. 

### 3.2. Antioxidant Activity of Brussels sprouts Raw and Subjected to Hydrothemal Treatments before and after In Vitro Digestion

**Figure 2:**
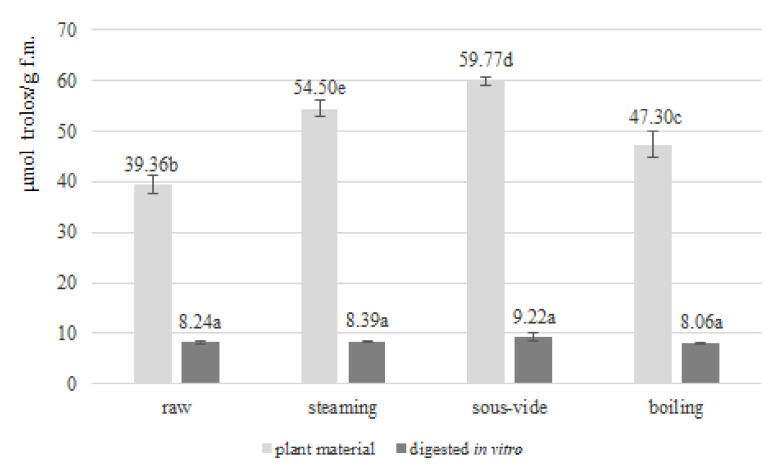
ABTS antioxidant activity (µmol trolox/g f.m.) in raw and thermally-treated *Brussels sprouts* before and after in vitro digestion. Means followed by the same letter (a,b,c,d,e) are not significantly different (*p* ≤ 0.05); f.m.—fresh matter.

**Figure 3:**
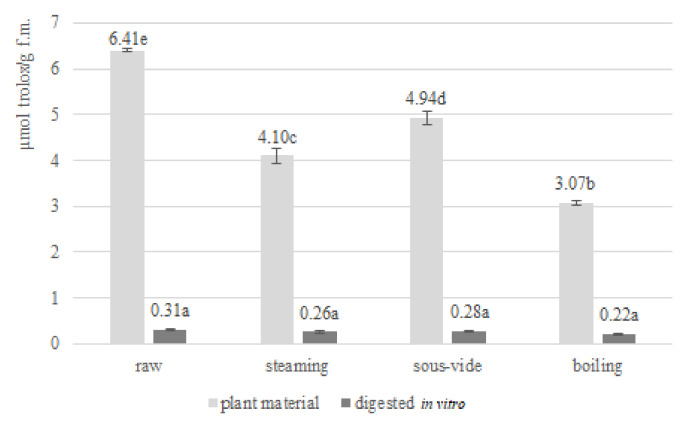
DPPH antioxidant activity (µmol trolox/g f.m.) in raw and thermally-treated *Brussels sprouts* before and after in vitro digestion. Means followed by the same letter (a,b,c,d,e) are not significantly different (*p* ≤ 0.05); f.m.—fresh matter.

The antioxidant activity of fruits and vegetables is gaining more and more interest each year. The pro-health effects of naturally occurring antioxidants have also been confirmed in *Brassica* vegetables [18]. The antioxidant activity was determined spectrophotometrically by measuring the ability of the antioxidant compounds present in the analysed *Brussels sprouts* to scavenge ABTS and DPPH free radicals. The antioxidant activity determined by the ABTS free radical assay of raw *Brussels sprouts* and those subjected to three hydrothermal treatments before and after in vitro digestion is presented in Figure 2. Raw *Brussels sprouts* indicated an antioxidant potential of 39.36 ± 1.75 (µmol trolox/g f.m.). This result is higher than the result obtained in *Brassica oleracea* var. *gemmifera* by other authors. Lower values of antioxidant activity determined by ABTS radicals in relation to *Brussels sprouts* were demonstrated in white and green rose cauliflower, broccoli and kale [5]. These differences may be due to different harvest dates and other species of *Brassica* vegetables analysed. The antioxidant activity determined by the DPPH free radical assay of raw *Brussels sprouts* and those subjected to three hydrothermal treatments before and after in vitro digestion is presented in Figure 3. Raw *Brassica oleracea* var. *gemmifera* demonstrated an antioxidant activity of 6.41 ± 0.03 (µmol trolox/g f.m.). This value is higher than in the cases of swede, Chinese cabbage, pakchoi, broccoli, cauliflower and cabbage detected by other authors and is lower than the DPPH activity measured in *Brussels sprouts*. These differences may result from different cultivation conditions, harvest time and species [19,32].

The antioxidant activity (ABTS radical) also, as in the case of total polyphenol concentration, increased with all hydrothermal treatments compared with raw *Brussels sprouts*. This increase may be due to an increase in the concentration of components with antioxidant activity as a result of damage to cell walls and membranes during thermal treatment allowing their availability for extraction prior to determination. In addition, new compounds with antioxidant activity such as Maillard reaction products, are formed during hydrothermal treatment. An inverse effect was observed in case of antioxidant activity (DPPH radical), where all thermal treatments caused a decrease in antioxidant potential. This reduction may result from plant tissue damage, temperature influence and enzyme activity. The differences between ABTS and DPPH values may result from the fact that measured antioxidant potential not only depend on the plant matrix analysed but also strongly depends on the type of methodology used and free radical chosen [33]. 

For both ABTS and DPPH, the highest antioxidant activity of all the applied methods was observed with the sous-vide technique (ABTS: 59.77 ± 0.75 µmol trolox/g f.m.; DPPH: 4.94 ± 0.15 µmol trolox/g f.m.). This due to the vacuum packaging limiting the contact between the plant material and the medium and the reduced thermal degradation of bioactive compounds due to lower process temperature. A lower activity than sous-vide was retained in the case of steaming (ABTS: 54.50 ± 1.51 µmol trolox/g f.m.; DPPH: 4.10 ± 0.17 µmol trolox/g f.m.), and the lowest was recorded during traditional boiling in water (ABTS: 47.30 ± 2.58 µmol trolox/g f.m.; DPPH: 3.07 ± 0.06 µmol trolox/g f.m.). This correlation may be directly related to the thermal degradation of bioactive compounds, their loss by leaching into the aqueous medium or the absorption of water by plant tissues during cooking. Moreover, the compounds with antioxidant activity are also prone to enzymatic degradation that occurs when bioactive raw materials are exposed to enzymes as a result of mechanical and thermal damage to the plant tissues. These results are consistent with those obtained by other authors, who also demonstrated higher antioxidant potential of sous-vide-treated vegetables compared with other methods [5,20]. Other authors identified boil-in-bag as a method that allows higher antioxidant activity than the sous-vide method for swedes; however, it was still the methods involving vacuum packaging that were more effective against bioactive compounds than conventional methods [19]. As in the case of polyphenols and antioxidant activity (FRAP and DPPH), a divergent effect of hydrothermal treatment methods in relation to plant morphological parts was demonstrated in another study. In the case of leaves of *Espigall del Garraf*, stems of broccoli cv. *Parthenon* and kale cv. *Crispa* sous-vide proved to be a more delicate processing method in relation to steaming allowing higher antioxidant activity, as in our study. An opposite correlation was observed in the case of inflorescences of broccoli cv. *Parthenon* and broccoli cv. Marathon [18]. Furthermore, ref. [22] has indicated that the sous-vide technique allows the highest level of antioxidant activity (with microwave and frying achieving the same result). 

The in vitro digestion procedure reduced the antioxidant activity of raw and thermally-treated *Brussels sprouts* (*p* ≤ 0.05). The type of hydrothermal treatment selected did not affect the level of antioxidant activity of *Brassica oleracea* var. *gemmifera* after in vitro digestion in case of both ABTS and DPPH radicals. The decrease in antioxidant activity (ABTS and DPPH) is consistent with the results presented by other authors. A model study of the effect of in vitro digestion (centrifugation, filtration and dialysis) on antioxidant activity demonstrated a decrease in activity for rosmarinic acid (24–36%), caffeic acid (12–19%) and no change in antioxidant capacity for rutin [24]. A similar correlation of the reduction of antioxidant activity (FRAP, DPPH, CUPRAC) for plant-processing by-products of black carrot was also observed during the gastric stage of in vitro digestion, while at further stages of digestion the results remained divergent indicating an increase or decrease depending on the method and raw material [31]. The reduction in antioxidant activity (ABTS and DPPH) observed in our study and in the work of other authors during the intestinal part of in vitro digestion can be explained by the structural reorganisation of some bioactive compounds during a change in pH to slightly alkaline. Moreover, these compounds gain the ability to react and bind with other components of the plant tissue matrix, which results in a reduction of their antioxidant potential [7]. Oppositely, for grape juice digested in vitro, only an increase in the antioxidant activity of ABTS was reported after switching from the gastric to the intestinal stage of in vitro digestion [26]. These discrepancies may be dictated by the species of analysed plant raw materials, the different characteristics, chemical structure and initial concentration of bioactive compounds, the initial antioxidant activity of the raw material, the methodology for the determination of antioxidant activity and the in vitro digestion procedure.

### 3.3. Polyphenolic Acids Concentration in Brussels sprouts Raw and Subjected to Hydrothemal Treatments before and after In Vitro Digestion

Polyphenolic acids represent the most numerous group of polyphenolic compounds present in vegetables, fruits, beverages, spices and cereal products. They demonstrate anti-inflammatory effects, prevent the formation of free radicals by blocking metal catalysis, and constitute a protective factor for cardiovascular diseases. They are characterised by anti-mutagenic and hypoglycaemic effects [34]. The concentration of individual polyphenolic acids in raw and hydrothermally-treated *Brussels sprouts* before and after in vitro digestion is shown in Table 1. Among the analysed polyphenolic acids, sinapinic acid (392.49 ± 0.52 mg/kg f.m.) and gallic acid (136.81 ± 0.33 mg/kg f.m.) had the highest contents, while syringic acid (5.26 ± 0.09 mg/kg f.m.), chlorogenic acid (10.14 ± 0.00 mg/kg f.m.), protocatechuic acid (13.43 ± 0.38 mg/kg f.m.) and 4-hydroxybenzoic acid (13.53 ± 0.09 mg/kg f.m.) had the lowest concentrations. No rosmarinic acid was detected in raw *Brassica oleracea* var. *gemmifera*. The thermal treatment had a statistically significant effect on the concentration of polyphenolic acids. In the case of traditional boiling in water, with the exception of protocatechuic acid, this method contributed to a reduction in the content of individual polyphenolic acids. This is directly related to the leaching of polyphenolic compounds into the aqueous medium. Furthermore, steaming contributed mainly to a reduction in the concentration of the analysed polyphenolic compounds, with the exceptions of 4-hydroxybenzoic acid, caffeic acid, sinapinic acid and rosmarinic acid. However, steaming allowed statistically higher levels of polyphenolic acids than traditional boiling in water, thus contributing to lower losses. Furthermore, other authors examining the steam cooking of *Brassica oleracea* var. *gemmifera* have reported a mainly reducing effect in relation to the concentration of polyphenolic acids [35]. 

In contrast to the previously described hydrothermal treatment methods, the sous-vide technique in the case of polyphenolic acids mostly increased their concentration with the exceptions of gallic acid, *p*-Coumaric acid and ferulic acid. Hydrothermal treatment contributes to the release of polyphenolic compounds contained in the apoplasm and vacuoles and those bound to plant cell wall components. In the case of traditional boiling in water, the released polyphenolic compounds are washed into the aqueous medium through direct contact between the plant material and the water, resulting in a high percentage of their loss. In the case of steam cooking, the acids can be oxidised, while the sous-vide technique, due to the use of vacuum packaging, protects the bioactive compounds from oxidation and leaching into the medium, and is thus the most effective treatment for most of the analysed polyphenolic acids among the techniques used. These results are consistent with those obtained by other authors, who also identified the sous-vide technique as the hydrothermal treatment allowing the highest stability of *p*-Coumaric, caffeic and gallic acids in broccoli, white and green rose cauliflower and *Brussels sprouts* [5]. Opposite to our own results, ref. [35] has also reported a decrease in the content of individual polyphenolic acids in the case of sous-vide, this may be due to both different conditions of the sous-vide technique and the fact that the authors analysed sous-vide samples during one-day, five-day and 10-day storage and not immediately after processing as in our study. This may have contributed to the softening of the plant tissue matrix, thus allowing oxidation of polyphenolic compounds by residual oxygen in the package. 

Following the in vitro digestion procedure, the concentration of individual polyphenolic acids was reduced (*p* ≤ 0.05) except for *p*-Coumaric acid in steamed *Brussels sprouts* and rosmarinic acid in raw *Brassica oleracea* var. *gemmifera*. Opposite to *Brussels sprouts* before the digestion (reduction of content) and after in vitro digestion, the content of polyphenolic acids after traditional boiling in water remained statistically comparable with raw *Brussels sprouts* or higher. Furthermore, in the case of steaming and sous-vide in most of the analysed polyphenolic components, a similar trend as in the case of traditional boiling in water was observed—an increase in the concentration of bioavailable polyphenolic acids. These results indicate an increase in the pool of bioavailable polyphenolic compounds usable by the consumer’s body. Oppositely to the analysed plant raw material samples, in the case of in vitro dialysates after digestion, steaming enabled in most cases the highest level of polyphenolic acid preservation. Maintaining the trend before the digestion procedure, traditional boiling in water contributed to the highest losses of bioavailable polyphenolic acids compared to raw *Brassica oleracea* var. *gemmifera*. Other authors have indicated a high stability of polyphenolic acids following in vitro gastric digestion, and a decrease in their concentration, as in our study, due to the influence of a slightly alkaline in vitro intestinal environment in the case of soy milk [36] and mango by-product snacks [37]. In plant-processing by-products of black carrot, a decrease in neochlorogenic acid, cryptochlorogenic acid and chlorogenic acid content was also observed during the gastric stage of in vitro digestion, which was exacerbated at further stages of digestion. Contrastingly, caffeic acid and ferulic acid mostly demonstrated an increase in bioavailability in opposition to undigested samples [31]. This variation in the results is probably dictated by both the different species of plant materials analysed, their degree of processing and the in vitro digestion methodology.

### 3.4. Flavonoids Concentration in Brussels sprouts Raw and Subjected to Hydrothemal Treatments before and after In Vitro Digestion

Flavonoids demonstrate carcinogenesis inhibitory activity due to their high scavenging of free radicals, resulting in the prevention of DNA damage that can lead to pathological conditions. In addition, they activate phase I and II of enzymes, enhancing the excretion of carcinogenic substances from human cells. It has been scientifically proven that the risk of cancer is twice as low in people whose diets are characterised by a high consumption of vegetables and fruits providing an adequate concentration of flavonoids, compared with people whose diets are poor in plant products [23]. The concentrations of individual flavonoids in raw and hydrothermally-treated *Brussels sprouts* before and after in vitro digestion are presented in Table 2. Of the flavonoids analysed, catechin (96.65 ± 1.22 mg/kg f.m.) and epicatechin (27.62 ± 0.28 mg/kg f.m.) had the highest content, while hispidulin (3.62 ± 0.05 mg/kg f.m.), hesperidin (6.43 ± 0.05 mg/kg f.m.) and acacetin (8.78 ± 0.05 mg/kg f.m.) had the lowest concentrations. No presence of isorhamnetins, apigenins, kaempferol and quercetins was detected in raw *Brussels sprouts*. However, thermal treatment (mainly steaming and sous-vide) resulted in the presence of these compounds in *Brassica oleracea* var. *gemmifera*.

Thermal treatment had a statistically significant effect on the concentration of flavonoids. Conventional boiling in water contributed to a decrease in flavonoid content in most cases except naringin, apigenin and hispidulin. Steaming mainly caused an increase in the concentration of these compounds with the exceptions of epicatechin, rutin, luteolin, hispidulin and acacetin. Sous-vide also mainly increased the concentration of individual flavonoids with the exceptions of catechin, epicatechin, rutin, luteolin, hispidulin and acacetin. However, in the case of flavonoids, and in opposition to polyphenolic acids, it was the steaming that made it possible to obtain a product with a higher concentration of the studied compounds than the sous-vide technique. These results are consistent with the results obtained in another study, in which the authors also identified steaming as a method allowing higher flavonoid levels than other hydrothermal treatments (sous-vide, boiling and microwaving) of broccoli (*Brassica oleracea* var. Avenger) and cauliflower (*Brassica oleracea* var. Alphina F1) grown in an organic system [38]. Furthermore, other authors have found a statistically significant reduction in flavonoid concentration when broccoli, kale, *Brussels sprouts* and white and green rose cauliflower were cooked conventionally in water [39]. Flavonol glycosides located in the upper layers of the hydrophilic plant tissue have a high degree of solubility in water, hence the highest losses are observed in the case of traditional cooking in water. Therefore, in order to achieve a higher retention of flavonoid bioactive components, hydrothermal processing methods should be applied that limit the contact of the plant material with the aqueous medium [39]. These observations and recommendations are in accordance with the obtained results.

After the in vitro digestion procedure, in most cases the concentration of flavonoids was reduced compared with the plant material not subjected to digestion (*p* ≤ 0.05). Apart from apigenin in the case of all hydrothermal treatments and hispidulin and quercetin in the case of steaming, the thermal treatment of *Brussels sprouts* increased the concentration of bioavailable flavonoids compared with raw *Brassica oleracea* var. *gemmifera*. As in the case of polyphenolic acids, steaming enabled the highest levels of flavonoids in digested *Brussels sprouts*, while sous-vide and conventional boiling in water produced lower concentrations of these compounds. Oppositely, other authors have found high flavonoid stability following in vitro application to the gastric digestion section. However, after changing the acidic gastric environment to a slightly alkaline intestinal environment at a further stage of digestion, they observed, as in their own studies, a decrease in the concentration of individual flavonoids in the case of grape juice [26] and soy milk [36]. There are also papers, however, which indicate a decrease in flavonoid content at both the gastric and intestinal stages of in vitro digestion as in the case of aqueous infusions from *Crithmum* *maritimum* L. and *Capparis* *spinosa* L. [31]. Furthermore, as in the case of polyphenolic acids, this variation in results is probably dictated by the different species of plant materials analysed, their degree of processing and the in vitro digestion methodology.

Flavonoids in both the analysed *Brassica oleracea* var. *gemmifera,* regardless of the selected processing method, and in the plant material that was not subjected to hydrothermal treatment were present in lower concentration than polyphenolic acids. Thus, *Brussels sprouts* are a better dietary source of polyphenolic acids than flavonoids. These results are in agreement with those obtained by other authors [35].

## 4. Conclusions

Cultivation and climatic conditions, soil type, post-harvest treatment and storage can affect the antioxidant potential of foods. In the case of vegetables, such as the analysed *Brussels sprouts* (*Brassica oleracea* var. *gemmifera*), that are consumed mainly after thermal treatment, the significant influence of hydrothermal food processing conditions should be considered when analysing the content of bioactive compounds. The study proved a statistically significant effect of the type of hydrothermal treatment on total polyphenol concentration, polyphenolic acid profile, flavonoid content and antioxidant activity. Traditional boiling in water was the least effective hydrothermal treatment for the bioactive components of *Brassica oleracea* var. *gemmifera* due to the leaching of nutrients and bioactive components into the aqueous medium. Sous-vide was the most effective method of hydrothermal treatment in terms of retention of polyphenolic compounds and high antioxidant activity, thus constituting a better alternative to steaming. This is due to the use of vacuum packaging limiting the contact of the raw material with water and to the strictly controlled temperature conditions (with the lowest process temperature among those applied). However, polyphenolic compounds are present in the food matrix in a form that prevents their direct utilisation by the human body, having to undergo a digestive process beforehand. During digestion, bioavailable bioactive compounds are released from the food matrix and are absorbed and assimilated. Their concentration, which was confirmed in our study by using an in vitro model, is different from the content in the plant material before digestion. The influence of the type of hydrothermal treatment on the concentration of bioavailable polyphenolic compounds (after in vitro digestion) remained preserved compared with the raw material not subjected to in vitro digestion (except for antioxidant activity); with sous-vide and steaming mainly enabling the highest concentration of bioavailable bioactive compounds and boiling in water contributing to the highest losses. The application of the in vitro digestion model enabled a more detailed characterisation of the concentration of individual bioactive compounds of *Brassica oleracea* var. *gemmifera* actually available for use by the human body.

## Figures and Tables

**Table 1:** Phenolic acids concentration (mg/kg f.m.) in raw and thermally-treated *Brussels sprouts* before and after in vitro digestion.

Phenolic Acid		Raw	Thermal Treatment		
			Steaming	Sous-Vide	Boiling
Gallic acid	**A**	136.81 ± 0.33 f	51.17 ± 0.18 c	69.13 ± 0.10 e	62.45 ± 1.46 d
	**B**	3.08 ± 0.00 a	14.71 ± 0.01 b	3.58 ± 0.01 a	3.50 ± 0.02 a
Chlorogenic acid	**A**	10.14 ± 0.00 f	4.89 ± 0.02 d	11.14 ± 0.11 g	7.76 ± 0.00 e
	**B**	0.44 ± 0.00 a	0.00 ± 0.00 c	0.70 ± 0.00 b	0.53 ± 0.00 ab
4-Hydroxybenzoic acid	**A**	13.53 ± 0.09 c	22.42 ± 0.77 d	26.18 ± 0.02 e	6.11 ± 0.22 b
	**B**	0.93 ± 0.01 a	2.05 ± 0.03 a	1.16 ± 0.00 a	1.24 ± 0.02 a
Caffeic acid	**A**	34.10 ± 0.00 d	51.31 ± 0.05 h	48.43 ± 0.05 g	27.83 ± 0.09 c
	**B**	8.53 ± 0.00 a	44.06 ± 0.03 f	37.94 ± 0.01 e	22.12 ± 0.03 b
Vanillic acid	**A**	24.66 ± 0.05 g	22.34 ± 0.02 f	25.71 ± 0.02 h	20.54 ± 0.37 e
	**B**	3.70 ± 0.00 a	15.55 ± 0.05 d	13.78 ± 0.01 c	8.48 ± 0.11 b
Syringic acid	**A**	5.26 ± 0.09 a	5.24± 0.03 a	6.66 ± 0.02 e	5.34 ± 0.00 a
	**B**	0.47 ± 0.00 c	1.34 ± 0.00 d	1.69 ± 0.02 b	1.64 ± 0.01 b
*p*-Coumaric acid	**A**	17.56 ± 0.00 g	5.32 ± 0.11 e	4.62 ± 0.02 d	4.96 ± 0.00 a
	**B**	4.84 ± 0.01 a	5.83 ± 0.01 f	3.70 ± 0.00 c	2.26 ± 0.00 b
Ferulic acid	**A**	36.07 ± 0.09 g	33.54 ± 0.08 f	28.86 ± 0.07 e	22.62 ± 0.03 a
	**B**	9.45 ± 0.01 b	27.22 ± 0.08 d	22.57 ± 0.01 a	16.99 ± 0.06 c
Sinapinic acid	**A**	392.49 ± 0.52 d	550.93 ± 1.25 h	500.39 ± 0.53 g	303.20 ± 0.03 c
	**B**	94.97 ± 0.11 a	453.90 ± 0.06 f	407.21 ± 0.30 e	267.24 ± 0.46 b
Rosmarinic acid	**A**	0.00 ± 0.00 a	1.05 ± 0.01 b	1.03 ± 0.01 b	0.00 ± 0.00 a
	**B**	0.68 ± 0.01 c	0.00 ± 0.00 a	0.00 ± 0.00 a	0.00 ± 0.00 a
Protocatechuic acid	**A**	13.43 ± 0.38 b	0.00 ± 0.00 a	85.00 ± 0.13 d	40.95 ± 0.87 c
	**B**	0.00 ± 0.00 a	0.00 ± 0.00 a	0.00 ± 0.00 a	0.00 ± 0.00 a

* Results are shown as mean ± standard deviation (SD); means followed by the same letter (a,b,c,d,e,f,g,h) in a row A and B for the same compound are not significantly different (*p* ≤ 0.05); A—values before in vitro digestion, B—values after in vitro digestion; f.m.—fresh matter.

**Table 2:** Flavonoids concentration (mg/kg f.m.) in raw and thermally-treated *Brussels sprouts* before and after in vitro digestion.

Flavonoid		Raw	Thermal Treatment		
			Steaming	Sous-Vide	Boiling
Catechin	**A**	96.65 ± 1.22 b	121.03 ± 0.03 f	12.30 ± 0.51 d	95.06 ± 2.79 b
	**B**	1.74 ± 0.97 c	74.27 ± 0.20 e	21.52 ± 0.02 a	24.04 ± 0.22 a
Epicatechin	**A**	27.62 ± 0.28 f	11.42 ± 0.01 b	5.24 ± 0.03 e	12.88 ± 0.40 c
	**B**	3.34 ± 0.01 d	12.10 ± 0.16 bc	8.55 ± 0.07 a	9.58 ± 0.09 a
Naringin	**A**	19.63 ± 0.00 c	23.69 ± 0.03 d	46.29 ± 0.09 f	25.94 ± 0.37 e
	**B**	0.68 ± 0.00 a	2.16 ± 0.00 b	1.24 ± 0.03 a	0.83 ± 0.03 a
Rutin	**A**	10.80 ± 0.09 f	4.50 ± 0.04 d	1.00 ± 0.05 a	9.25 ± 0.06 e
	**B**	1.01 ± 0.01 a	6.58 ± 0.00 bc	6.27 ± 0.20 b	6.82 ± 0.00 c
Hesperidin	**A**	6.43 ± 0.05 c	23.32 ± 0.09 h	20.93 ± 0.03 g	5.21 ± 0.31 b
	**B**	0.68 ± 0.01 a	8.32 ± 0.02 d	18.15 ± 0.01 f	10.15 ± 0.00 e
Myricetin	**A**	0.00 ± 0.00 a	0.00 ± 0.00 a	2.01 ± 0.01 e	0.00 ± 0.00 a
	**B**	0.60 ± 0.01 b	2.70 ± 0.00 f	1.29 ± 0.01 d	1.11 ± 0.01 c
Quercetin	**A**	0.00 ± 0.00 a	0.00 ± 0.00 a	1.85 ± 0.01 d	0.00 ± 0.00 a
	**B**	0.58 ± 0.00 c	0.00 ± 0.00 a	1.14 ± 0.01 b	1.12 ± 0.01 b
Luteolin	**A**	15.31 ± 0.09 h	5.13 ± 0.00 c	2.60 ± 0.03 a	9.06 ± 0.12 g
	**B**	4.35 ± 0.02 b	7.90 ± 0.02 e	7.08 ± 0.02 d	8.26 ± 0.00 f
Kaempferol	**A**	0.00 ± 0.00 a	2.23 ± 0.00 e	2.81 ± 0.01 f	0.00 ± 0.00 a
	**B**	0.52 ± 0.00 b	2.94 ± 0.02 g	1.91 ± 0.00 d	1.48 ± 0.02 c
Apigenin	**A**	0.00 ± 0.00 a	1.72 ± 0.01 c	1.52 ± 0.08 bc	14.86 ± 0.65 d
	**B**	0.39 ± 0.00 ab	0.00 ± 0.00 a	0.00 ± 0.00 a	0.00 ± 0.00 a
Isorhamnetin	**A**	0.00 ± 0.00 a	1.56 ± 0.02 b	0.79 ± 0.03 d	0.00 ± 0.00 a
	**B**	0.28 ± 0.01 c	1.37 ± 0.01 e	1.63 ± 0.00 b	1.45 ± 0.00 e
Hispidulin	**A**	3.62 ± 0.05 e	1.43 ± 0.02 b	1.32 ± 0.01 b	6.48 ± 0.03 f
	**B**	0.29 ± 0.00 d	0.00 ± 0.00 c	0.61 ± 0.00 a	0.59 ± 0.01 a
Acacetin	**A**	8.78 ± 0.05 g	5.43 ± 0.01 f	2.44 ± 0.01 e	0.00 ± 0.00 b
	**B**	0.70 ± 0.00 a	1.54 ± 0.00 d	0.76 ± 0.00 a	1.09 ± 0.02 c

* Results are shown as mean ± standard deviation (SD); means followed by the same letter (a,b,c,d,e,f,g,h) in a row A and B for the same compound are not significantly different (*p* ≤ 0.05); A—values before in vitro digestion, B—values after in vitro digestion; f.m.—fresh matter.

## Data Availability

The data are contained within the article.
